# Pigment Epithelium-Derived Factor-Loaded PEGylated Nanoparticles as a New Antiangiogenic Therapy for Neovascularization

**DOI:** 10.1155/2022/1193760

**Published:** 2022-04-22

**Authors:** Feng Zhao, Wenlei Fei, Zhouyue Li, Hanyang Yu, Lei Xi

**Affiliations:** ^1^Guangdong Provincial People's Hospital, Guangdong Academy of Medical Sciences, Guangzhou, Guangdong, China; ^2^State Key Laboratory of Ophthalmology, Zhongshan Ophthalmic Center, Sun Yat-sen University, Guangzhou, Guangdong, China

## Abstract

**Background:**

Pathological neovascularization, which involves a disruption in the balance between angiogenic and antiangiogenic factors under pathological conditions, is the basis of many intraocular diseases. Pigment epithelium-derived factor (PEDF) is a potent natural, endogenous inhibitor of neovascularization because of its antiangiogenic and neuroprotective benefits. However, its application is restricted by its instability and short half-life. The present study is aimed at investigating the cytotoxicity and antiangiogenic effects of PEDF-loaded PEGylated nanoparticles (NP-PEG-PEDF) on high glucose-stimulated human umbilical vein endothelial cells (HUVECs).

**Methods:**

In this study, NP-PEG-PEDF were fabricated using the multiple emulsion method for the first time. HUVECs were cultured in a high concentration of glucose (30 mmol/L D-glucose), simulating diabetic conditions. The antiangiogenic effects of vascular endothelial growth factor (VEGF), pure PEDF, and NP-PEG-PEDF on proliferation, migration, and tube formation were evaluated. VEGF secretion in high glucose-stimulated HUVECs was further tested in vitro.

**Results:**

NP-PEG-PEDF exhibited low cytotoxicity in HUVECs. Our results indicated that in vitro, NP-PEG-PEDF attenuated diabetes-induced HUVEC proliferation, migration, and tube formation and suppressed VEGF secretion. The apoptosis of diabetes-induced HUVECs occurred in a dose-dependent manner, which showed a statistically significant difference compared with the PEDF treatment group.

**Conclusion:**

Our study is the first to demonstrate that NP-PEG-PEDF exert antiangiogenic effects on high glucose-stimulated HUVECs and have the potential to alleviate microvascular dysfunction. These data suggest that the NP-PEG-PEDF delivery system may offer an innovative therapeutic strategy for preventing neovascularization of the fundus.

## 1. Introduction

Pathological neovascularization (NV) in the fundus, such as choroidal NV and retinal NV, is the basic pathological changes in many intraocular diseases, such as age-related macular degeneration (AMD) [1], pathologic myopia [2], and proliferative diabetic retinopathy (PDR) [3]. The reason for the formation of NV remains unclear, but it involves a disruption in the balance between angiogenic and antiangiogenic factors under pathological conditions, such as hypoxia and inflammation [4, 5]. Over the last decade, inhibition of vascular endothelial growth factor A has emerged as a treatment modality for choroidal neovascularization, despite the fact that a considerable number of patients are refractory to anti-VEGF therapy [6]. Additionally, the finding that anti-VEGF therapy may also affect the physiological blood vessels and tissue has raised concerns regarding the long-term adverse effects of this therapy [7]. Developing new antiangiogenic agents to achieve enhanced efficacy and decreased side effects is another option to address these issues.

Pigment epithelium-derived factor (PEDF), which is secreted by the retinal pigment epithelium (RPE) has antiangiogenic, anti-vasopermeability, and antineurotrophic functions, and it is a prospective biomarker that may inhibit the development of diabetic retinopathy (DR) [8–10]. According to current studies, PEDF is regarded as a promising ocular protecting agent capable of blocking angiogenesis in the choroid and is an essential contributor to maintaining the retinal vascularity status through its antioxidative properties [11–13]. However, difficulty in sustaining it in an active state and lack of an appropriate delivery system limit its application as an antiangiogenic drug in clinical practice.

Polyethylene glycol (PEG) is a type of surface-modifying agent that helps to achieve mucoadhesive, stable, and stealth nanoparticles [14–16]. It has been reported that an optimized PEGylated nanostructured lipid carrier (NLC) could be physically and chemically stable for at least one month as an aqueous dispersion [17]. In this study, we formulated PEDF-loaded PEGylated nanoparticles (NP-PEG-PEDF) and evaluated their cytotoxicity and antiangiogenic effects in vitro with high glucose-treated human umbilical vein endothelial cells (HUVECs). Our results presented a potential strategy for the treatment of NV in the fundus via an NP-PEG-PEDF drug delivery system.

## 2. Materials and Methods

### 2.1. Cell Culture

HUVECs were obtained from the American Tissue Culture Collection (ATCC USA) and cultured in Dulbecco's Modified Eagle Medium (DMEM) with 10% fetal bovine serum (FBS; Hyclone, Grand Island, NY, USA) in a 37°C humidified incubator with 5% CO_2_ atmosphere.

### 2.2. Preparation of NP-PEG-PEDF

Lecithin, cholesterol, DSPE-PEG-FA (a PEG derivative containing folic acid), and PEDF were mixed in a chloroform/methanol solution at a mass ratio of 1.7 : 2 : 1.7 : 1. The organic solvent was removed by rotary evaporation, and a thin film of phospholipids was formed on the walls of the round-bottomed flask. Ultrapure water was added to the mixture. A VCX130 ultrasonic crusher was used to ultrasonicate the mixture for 2 min at a frequency of 20 kHz and a power of 130 W to hydrate the film. The nanoparticles were filtered successively through 200 nm and 100 nm polycarbonate filters five times each, followed by three rounds of Amicon Ultra-4 centrifugation (Millipore, USA).

### 2.3. Characterization of NP-PEG-PEDF

The NP size (diameter, nm), polydispersity index, and surface charge (zeta potential, mV) were measured by quasielastic laser light scattering using a Zeta PALS dynamic light scattering (DLS) detector (Beckman Coulter, San Diego, USA) at 25°C. The dispersion of NPs was diluted to suitable concentration by ultrapure water before measurement. The morphology of NPs was observed by transmission electron microscopy (TEM). NP suspensions (2 mg/mL) were stained with 2% (*w*/*v*) phosphotungstic acid, and then, they were dropped on a 200-mesh copper grid coated with carbon, dried at room temperature, and observed by a TEM instrument at 100 kV (JEOL-1230, Japan).

### 2.4. HUVEC Viability Assay

HUVECs were used to detect the angiogenic effect of VEGF and the antiangiogenic effects of NP-PEG-PEDF and pure PEDF in vitro using a 3-(4,5-dimethylthiazol-2-yl)-2,5-diphenyltetrazolium (MTT) cell proliferation kit (Sangon, Shanghai, China) assay. HUVECs were combined in DMEM with a high-glucose concentration (30 mmol/L) at a density of 5 × 10^3^ cells per well in 96-well plates overnight without FBS. HUVECs were incubated with VEGF (20 ng/mL) or different concentrations of NP-PEG-PEDF or PEDF (10, 100, and 1000 ng/mL) for 24, 48, and 72 h. After adding the MTT reagent, the cells were incubated at 37°C for another 4 h. The medium was aspirated carefully from each well, and 150 *μ*L of DMSO (Sigma, St. Louis, MO) was added to dissolve formazan, the end product, and the absorbance was read at a wavelength of 540 nm using a plate reader. Cells treated with medium only were set as 100%, and the other experimental groups were calculated using the following formula: viability (%) = (mean optical density [OD] of cells of the experimental group–mean OD of blank)/(mean OD of cells of the control group–mean OD of blank).

To study the angiogenic inhibitory effects of NP-PEG-PEDF under the stimulation of VEGF, 20 ng/mL VEGF was added to the culture medium. PEDF and NP-PEG-PEDF at the same varying concentrations as tested above were incubated for 24, 48, and 72 h, as stated previously. Each experiment was performed in six wells and repeated at least three times.

### 2.5. Flow Cytometry Analysis of HUVEC Apoptosis

Apoptosis was measured using the FITC Annexin V Apoptosis Detection Kit (Beyotime Technology Ltd. Co., Shanghai, China), according to the manufacturer's instructions. In brief, HUVECs were seeded into 6-well plates and incubated with DMEM with high glucose (30 mmol/L) containing 10% FBS, VEGF (20 ng/mL), NP-PEG-PEDF (10, 100, and 1000 ng/mL), or PEDF (10, 100, and 1000 ng/mL) for 24, 48, and 72 h. The cells were then collected using 0.25% trypsin and stained with propidium iodide (PI) and annexin V for 30 min at 37°C. The early (annexin V^+^/PI^−^) and late (annexin V^+^/PI^+^) apoptotic cells were sorted by fluorescence-activated cell sorting (FACS) (BD Biosciences, San Diego, CA).

### 2.6. Scratch Wound Healing Assay

HUVECs (1 × 10^5^ cells per well) were seeded into 12-well plates and treated with DMEM containing 10% FBS. Once the cells reached approximately 80% confluence, the monolayer was scratched using a sterile p200 pipette tip. Unattached cells were removed by gentle washing. Subsequently, the attached cells were treated with DMEM with high glucose (30 mmol/L) containing 2% FBS and supplemented with VEGF (20 ng/mL), NP-PEG-PEDF (10 ng/mL and 100 ng/mL), or PEDF (10 ng/mL and 100 ng/mL) for 24 h. Cells were imaged at 0 h and 24 h under an inverted microscope. The following formula was used to calculate the wound closure ratio: wound closure (%) = (*A*_0_ − *A*_r_)/*A*_0_ × 100, where *A*_0_ represents the area of the initial wound area and *A*_r_ represents the remaining wound area at 24 h.

### 2.7. Tube Formation Study

The antiangiogenic potentials of VEGF, NP-PEG-PEDF, and PEDF were tested using a tube formation assay under high glucose (30 mmol/L). Aliquots (150 *μ*L) of Matrigel (BD Biosciences) solution were poured into 24-well plates and incubated at 37°C for 30 min in a 5% CO_2_ incubator. HUVECs treated with VEGF (20 ng/mL), NP-PEG-PEDF (10 ng/mL and 100 ng/mL), PEDF (10 ng/mL and 100 ng/mL), or controls were seeded on Matrigel and cultured in DMEM for 8 h. The networks in Matrigel from five randomly chosen fields were counted and photographed under a microscope. All experiments were performed in triplicate.

### 2.8. VEGF Detection by Enzyme-Linked Immunosorbent Assay

HUVECs were seeded into 96-well plates (1 × 10^4^ per well) with high glucose (30 mmol/L) and incubated at 37°C overnight. After removing the DMEM, NP-PEG-PEDF (10 ng/mL and 100 ng/mL) or PEDF (10 ng/mL and 100 ng/mL) were added to the wells. After 48 and 72 h of incubation, the supernatant of the cell culture was harvested, and the cellular debris was removed by centrifugation. VEGF protein secreted by HUVECs in the culture medium was measured using a VEGF ELISA kit purchased from BOSTER (Wuhan, China) according to the manufacturer's instructions.

### 2.9. Statistical Analysis

Data analysis was performed using STATA statistical software (version 15.0; StataCorp LLC). All data are presented as mean ± SEM and were evaluated for normality of distribution. Differences were evaluated using analysis of variance, followed by the Student-Newman-Keuls test for multiple comparisons and the Student's *t*-test for pairwise comparisons. Differences were considered statistically significant at *p* < 0.05.

## 3. Results

### 3.1. Characterization of NP-PEG-PEDF

**Figure 1:**
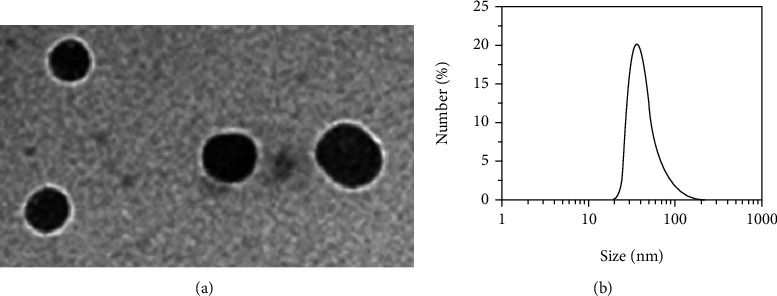
(a) Transmission electron microscopy image of NP-PEG-PEDF. (b) Size distribution of NP-PEG-PEDF.

Figures 1(a) and 1(b) show the transmission electron microscopy (TEM) image and size distribution of NP-PEG-PEDF, and the NPs were generally spherical in shape with good monodispersity. The zeta potential and hydrodynamic sizes of NP-PEG-PEDF were −11.7 ± 0.87 mV and 44.85 ± 4.78 nm with a polydispersity of 0.183, respectively. The drug encapsulation efficiency (EE) and drug loading efficiency (LE) of the NP-PEG-PEDF were observed at 46.25% and 7.23% which was crucial for clinical application.

### 3.2. Effect of NP-PEG-PEDF on HUVEC Proliferation

**Figure 2:**
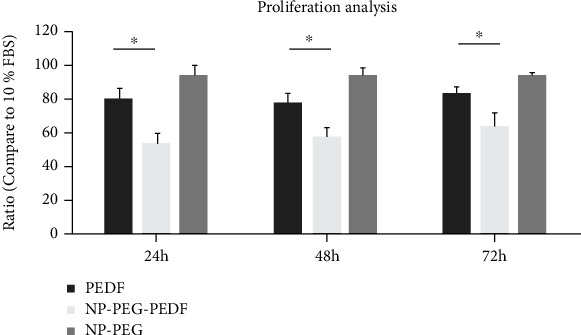
Effects of PEDF and NP-PEG-PEDF on the proliferation of HUVECs in the general culture medium under high glucose (30 mmol/L) at 24, 48, and 72 h. The effective concentrations (1000 ng/mL) at different time points are shown. ^∗^*p* < 0.05.

**Figure 3:**
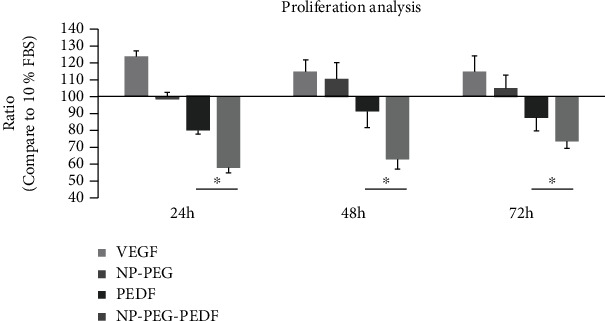
Effects of PEDF and NP-PEG-PEDF on the proliferation of HUVECs with VEGF stimulation under high glucose (30 mmol/L) at 24, 48, and 72 h. The effective concentrations (1000 ng/mL) at different time points are shown. ^∗^*p* < 0.05.

An HUVEC proliferation study was used to evaluate the antiangiogenic effects of NP-PEG-PEDF and pure PEDF in vitro under high glucose (30 mmol/L). HUVECs were incubated for 24, 48, and 72 h at various concentrations (10, 100, and 1000 ng/mL). As shown in Figure 2 and Table 1, the NP-PEG-PEDF-treated groups were statistically different from the PEDF-treated groups at all concentrations and time points. To further evaluate the inhibitory effect of NP-PEG-PEDF on VEGF stimulation, we added 20 ng/mL VEGF to the cell culture medium and found that NP-PEG-PEDF also exhibited better inhibitory effects on HUVEC proliferation (Figure 3 and Table 2).

### 3.3. Effect of NP-PEG-PEDF on HUVEC Apoptosis

**Figure 4:**
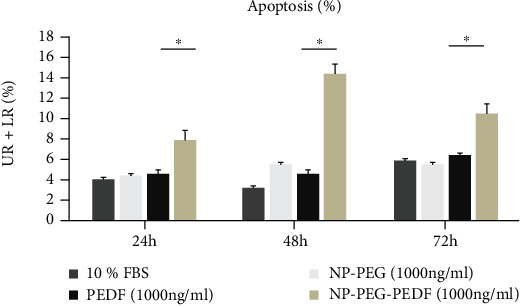
Effects of PEDF and NP-PEG-PEDF on HUVEC apoptosis under high glucose (30 mmol/L). DMEM+10% FBS control was set to 100%. ^∗^*p* < 0.05.

FACS was used to evaluate the effects of early and late apoptosis. As shown in Table 3 and Figure 4, after incubation with PEDF and NP-PEG-PEDF combined with 10% FBS under high glucose (30 mmol/L) for 24, 48, and 72 h, the early and late apoptotic HUVECs showed significant differences in the 10, 100, and 1000 ng/mL-treated groups, with the percentages of apoptotic cells (UR+LR) being significantly higher than those in PEDF-treated cells (*p* < 0.05).

LL: lower left; LR: lower right; UL: upper left; UR: upper right.

### 3.4. Effect of NP-PEG-PEDF on HUVEC Migration

**Figure 5:**
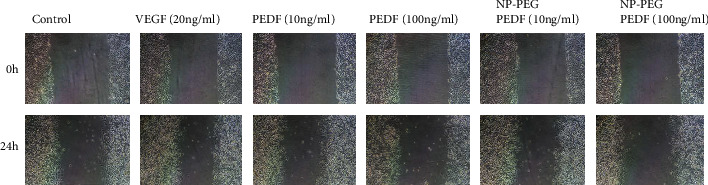
Cell scratch test results. Effects of PEDF and NP-PEG-PEDF on HUVEC scratch wound healing under high glucose (30 mmol/L).

**Figure 6:**
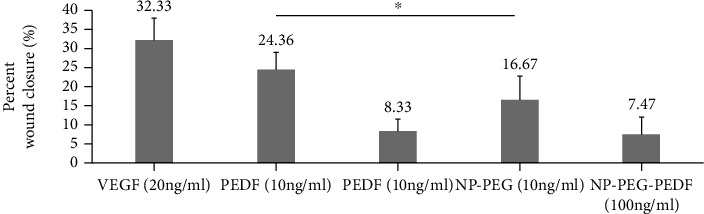
Statistical results of the percent wound closure (%) under different treatments. ^∗^*p* < 0.05.

HUVEC migration was assessed using a cell scratch test under high glucose (30 mmol/L). As shown in Figures 5 and 6, the percent wound closure of the membrane in the NP-PEG-PEDF-treated HUVEC group (10 ng/mL) was significantly lower than that in the PEDF (10 ng/mL) and VEGF (20 ng/mL)-treated groups (*p* 0.05).

### 3.5. Effect of NP-PEG-PEDF on HUVEC Tube Formation

**Figure 7:**
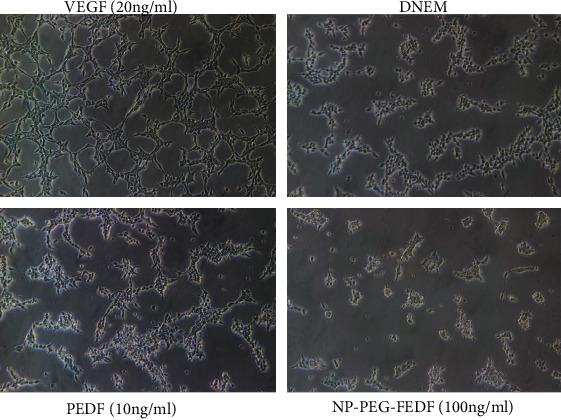
Effects of PEDF and NP-PEG-PEDF on HUVEC tube formation. NP-PEG-PEDF-treated HUVECs presented round morphology and showed no cell branches attempting to form networks.

**Figure 8:**
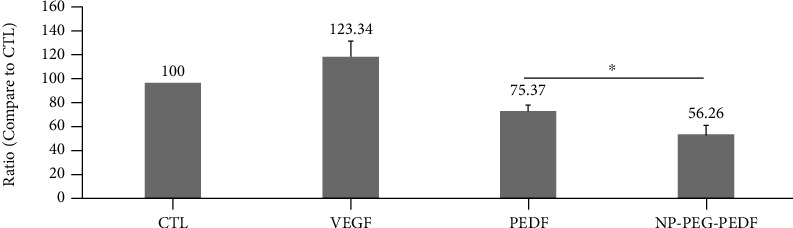
Statistical results of the length of tube formation under different treatments. DMEM+10% FBS control was set to 100%. ^∗^*p* < 0.05.

In our study, both PEDF- and NP-PEG-PEDF-treated cells exhibited an impaired capacity to form a regular network, and as expected, HUVECs could not form hollow lumens in either group (Figures 7 and 8). Although there was no significant difference between the NP-PEG-PEDF- and PEDF-treated groups (10 ng/mL) in terms of lumen formation, the NP-PEG-PEDF-treated HUVECs showed statistical differences in length compared with the PEDF-treated groups and presented round morphology with no branches at all, whereas the PEDF-treated group had a tendency to form tubes.

### 3.6. Effect of NP-PEG-PEDF on HUVECs in terms of the Reduction of VEGF Secretion

**Figure 9:**
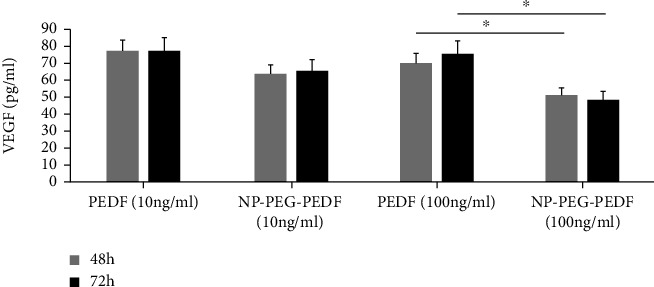
Effects of PEDF and NP-PEG-PEDF treatment on VEGF secretion in HUVECs. The NP-PEG-PEDF-treated group (100 ng/mL) exhibited significant suppression in VEGF secretion at both 48 and 72 h (*p* < 0.05).

As shown in Figure 9 and Table 4, after treatment for 48 and 72 h, VEGF was downregulated in the PEDF and NP-PEG-PEDF groups under high glucose (30 mmol/L). The NP-PEG-PEDF (100 ng/mL)-treated cells showed less VEGF secretion than the PEDF group at two time points (*p* < 0.05).

## 4. Discussion

Retinal or choroidal NV is the congenerous pathologic basis of several ocular diseases of the fundus, including PDR and AMD. The current research has shown that glucose metabolism influences the balance of proangiogenesis and antiangiogenesis processes involved in the progression of DR [18, 19]. Therefore, antiangiogenic treatment, especially intravitreal injection of anti-VEGF antibodies, is still the most effective therapeutic strategy throughout the world. However, its potent side effects, such as endophthalmitis, intraocular inflammation, ocular hemorrhage, retinal detachment, thrombosis, and cardiovascular accidents, drive us to look for new alternatives [20–22]. PEDF has been demonstrated to be an important therapeutic adjunct in the management of sight-threatening diseases caused by increased vascular permeability.

PEDF, a 50 kDa endogenously secreted glycoprotein [23], is a versatile protective angiogenic inhibitor for several ocular diseases [24–26]. It is expressed in many different cell types in fetal and adult eyes, including the cells of the cornea, lens, and retina. It is a novel agent that can be helpful in inhibiting the occurrence of new fragile blood vessels in the retina [27, 28]. There are many proposed mechanisms by which PEDF may decrease neovascularization and abate neovascular permeability: through inhibition of the VEGF pathway by downregulating mitogen-activated protein kinase-mediated hypoxia-inducible factor 1 activation [29], by enhancing the *γ*-secretase-dependent cleavage of the C-terminus phosphorylation in VEGF receptor 1 [30, 31], and activating apoptosis by regulating multiple pathways of p38 followed by cleavage of caspase-dependent [32, 33].

In addition, PEDF has nutritional and protective effects on nerves. PEDF can prevent neuronal apoptosis by upregulating the expression of B cell lymphoma 2 (Bcl-2) protein, when oxidative stress damage occurs in PDR [34, 35]. Increased PEDF can decrease H_2_O_2_-induced disruption of mitochondrial function and cell death in RPE cells by reducing oxidative stress [36]. Moreover, in vivo and in vitro studies revealed that PEDF neuroprotection was achieved by stabilizing photoreceptor degeneration by decreasing intracellular calcium and suppressing apoptotic and inflammatory pathways [37, 38]. In addition, PEDF can distinguish neovascular endothelial cells from normal vascular endothelial cells through Fas-FasL receptors, accomplish selective destruction of abnormal new blood vessels without damaging the normal established retinal blood vessels, and protect and maintain the status of healthy ocular tissues [12, 34, 35].

Because of its antiangiogenic and neuroprotective benefits, PEDF has been used to treat ocular NV. Investigators have evaluated virus-coded PEDF administered by intravitreous or subretinal injection in choroidal NV animal models. They found that overexpression of PEDF can inhibit neovascularization of the choroid and improve the survival of photoreceptors [39–42]. Bai et al. explored PEGylated-PEDF to prevent angiogenesis in HUVECs and in vivo [43]. The results revealed that PEGylated PEDF inhibited HUVEC proliferation, migration, and tube formation in a dose-dependent manner and decreased retinal neovascularization by intravitreous injection in an oxygen-induced retinopathy mouse model. However, there have not been any studies in diabetic models. The instability and short half-life of PEDF restrict its application. In recent years, nanoparticulate dosage forms have emerged as a promising ocular platform to deliver poorly water-soluble compounds because of their enhanced precorneal retention and improved penetration into the ocular tissues [16]. Additionally, nanomedicine has its own properties, including a high surface-to-volume ratio and favorable physicochemical characteristics [44]. PEG is commonly used for nanoparticle modification. The United States Food and Drug Administration has approved the use of PEG in drug modification for several years because of its low toxicity, low cost, and increased solubility [45]. However, it has not been determined if the processes of PEGylation and forming a PEDF-loaded nanostructured lipid altered the stability and toxic activity of PEDF.

In the current study, we developed NP-PEG-PEDF for the first time and evaluated its cytotoxicity and antiangiogenic effects in high-glucose cultured HUVECs. Our results showed that NP-PEG-PEDF inhibited HUVEC proliferation, migration, tube formation, and VEGF secretion and induced HUVEC apoptosis in a dose-dependent manner under diabetic conditions.

## 5. Conclusion

In summary, our study found, for the first time, that PEDF-loaded PEGylated nanoparticles effectively inhibited NV in high glucose-stimulated HUVECs in vitro. Because of the neurotrophic and antiangiogenic effects of PEDF, it may be utilized to make a more effective therapeutic treatment of retinal or choroidal NV in the future. Our data suggest that the PEGylated PEDF delivery system could be an extremely promising long-term treatment for PDR or AMD. NP-PEG-PEDF appears to be a safe and effective drug delivery system, and its use may prove to be an innovative approach for future therapeutic strategies against pathologically increased vascular permeability involved in several ocular diseases.

## Figures and Tables

**Table 1:** Summary of proliferation effects with PEDF and NP-PEG-PEDF-treated HUVECs under high glucose (30 mmol/L).

Time points		10%FBS	NP-PEG	PEDF			NP-PEG-PEDF		
			1000 ng/mL	1000 ng/mL	100 ng/mL	10 ng/mL	1000 ng/mL	100 ng/mL	10 ng/mL
24 h	Mean	1	94.27	80.33	77.34	69.15	54.19	45.83	40.34
	SEM		5.41	5.90	2.97	7.25	4.39	4.33	8.92
48 h	Mean	1	91.49	78.26	79.23	80.22	58.13	67.12	61.29
	SEM		6.87	3.69	4.89	5.32	5.02	3.71	10.23
72 h	Mean	1	91.12	83.89	86.13	88.3	64.29	63.12	59.56
	SEM		4.23	4.97	5.65	7.21	3.27	3.92	13.21

**Table 2:** Summary of proliferation effects of PEDF and NP-PEG-PEDF combined with VEGF-treated HUVECS under high glucose (30 mmol/L).

Time points		10% FBS	VEGF (20 ng/mL)	NP-PEG	PEDF			NP-PEG-PEDF		
				1000 ng/mL	1000 ng/mL	100 ng/mL	10 ng/mL	1000 ng/mL	100 ng/mL	10 ng/mL
24 h	Mean	1	123.61	99.19	79.83	74.57	84.48	57.32	59.48	62.07
	SEM		2.98	3.14	1.67	3.54	3.2	2.3	2.89	1.41
48 h	Mean	1	114.65	110.37	91.19	96.11	97.12	63.12	68.12	62.45
	SEM		6.56	9.24	7.23	5.29	5.81	4.32	5.27	6.82
72 h	Mean	1	115.21	105.25	87.68	90.58	91.03	73.33	79.71	87.82
	SEM		7.89	7.48	6.22	2.73	4.14	6.56	3.59	4.23

**Table 3:** Summary of flow cytometry data of apoptosis measured by annexin V and PI.

Time point	% of cells	10% FBS		NP-PEG		PEDF						NP-PEG-PEDF					
				1000 ng/mL		1000 ng/mL		100 ng/mL		10 ng/mL		1000 ng/mL		100 ng/mL		10 ng/mL	
		Mean	SEM	Mean	SEM	Mean	SEM	Mean	SEM	Mean	SEM	Mean	SEM	Mean	SEM	Mean	SEM
24 h	UL	0.87	0.09	2.29	0.11	2.34	0.15	2.51	0.17	1.65	0.17	1.27	0.07	1.67	0.1	2.26	0.15
	UR	2.16	0.06	2.36	0.17	2.23	0.14	2.43	0.13	1.89	0.19	2.91	0.13	3.23	0.16	2.15	0.22
	LL	95	0.21	93.17	0.23	93.02	0.34	93.33	0.32	94.11	0.39	90.84	0.21	91.12	0.24	91.44	0.23
	LR	1.97	0.12	2.18	0.09	2.41	0.21	1.73	0.09	1.33	0.11	4.98	0.19	3.98	0.22	2.01	0.18
	UR+LR	4.13	0.14	4.54	0.13	4.64	0.23	4.16	0.11	3.22	0.23	7.89	0.26	7.21	0.29	4.16	9.25
48 h	UL	1.39	0.14	1.93	0.07	2.34	0.11	3.44	0.13	2.66	0.21	2.43	0.11	2.89	0.14	2.87	0.15
	UR	1.12	0.13	1.55	0.12	1.56	0.09	1.54	0.09	1.53	0.15	3.97	0.21	2.93	0.24	2.33	0.51
	LL	95.33	0.23	92.54	0.34	92.98	0.38	90.34	0.2	92.25	0.42	83.17	0.34	82.84	0.37	88.21	0.31
	LR	2.16	0.14	3.98	0.25	3.12	0.21	4.68	0.19	2.69	0.45	10.43	0.21	11.34	0.24	4.03	0.17
	UR+LR	3.28	0.33	5.53	0.31	4.68	0.13	6.22	0.23	4.22	0.36	14.4	0.31	14.27	0.34	6.36	0.35
72 h	UL	2.11	0.28	1.91	0.09	1.93	0.11	1.98	0.12	2.32	0.15	2.33	0.11	2.07	0.14	3.00	0.11
	UR	2.03	0.31	2.03	0.11	2.57	0.21	2.63	0.21	2.36	0.13	3.78	0.13	3.13	0.16	3.32	0.24
	LL	91.99	0.26	94.16	0.35	91.52	0.32	92.07	0.34	91.19	0.33	87.12	0.29	87.49	0.32	87.29	0.52
	LR	3.87	0.25	1.9	0.07	3.98	0.08	3.32	0.12	3.31	0.23	6.77	0.13	7.31	0.16	3.21	0.37
	UR+LR	5.9	0.17	3.93	0.11	6.55	0.19	5.95	0.16	5.67	0.28	10.55	0.25	10.44	0.28	6.53	0.48

**Table 4:** Summary of VEGF secretion (pg/mL) in PEDF- and NP-PEG-PEDF-treated HUVECs.

Time points		10% FBS	PEDF		NP-PEG-PEDF	
			100 ng/mL	10 ng/mL	100 ng/mL	10 ng/mL
48 h	Mean	1	70.05	76.86	50.23	63.75
	SEM		3.65	6.79	2.76	4.69
72 h	Mean	1	75.81	77.91	47.92	65.78
	SEM		6.34	7.23	4.23	5.12

## Data Availability

The data used to support the findings of this study are available from the corresponding author upon request.
